# A HSP60-targeting peptide for cell apoptosis imaging

**DOI:** 10.1038/oncsis.2016.14

**Published:** 2016-02-29

**Authors:** S Yang, J Meng, Y Yang, H Liu, C Wang, J Liu, Y Zhang, C Wang, H Xu

**Affiliations:** 1 Institute of Basic Medical Sciences, Chinese Academy of Medical Sciences and Peking Union Medical College, Beijing, PR China; 2 CAS Key Laboratory for Biomedical Effects of Nanomaterials and Nanosafety, National Center for Nanoscience and Technology, Beijing, PR China; 3 Institute of Vascular Medicine, Peking University, Third Hospital, Beijing, PR China

## Abstract

Apoptosis has a critical role in both physiological and pathological processes, and therefore probes that enable direct and fast visualization for apoptosis *in vitro* and *in vivo* have great significance for evaluation of therapeutic effects, disease monitoring and drug screening. We report here a novel apoptotic marker heat shock protein 60 (HSP60)-based apoptosis imaging probe, P17. In this study, we show that P17 can label multiple drug-induced apoptotic cells *in vitro*, and the difference in binding intensities between apoptotic and viable cells by fluorescent P17 is more than 10-fold in six cell lines measured by flow cytometry and proportional to the apoptotic level of the cells. We further visualized the apoptosis in the subcutaneous tumor of mice by vein injection of P17 using *in vivo* fluorescent imaging. P17 was identified to bind specifically to HSP60 accumulated in apoptotic cells by pull-down experiments and mass spectrometry. Furthermore, the P17 binding was correlated with the apoptotic feature of phosphatidylserine (PS) exposure and caspase-3 activation. We also clarify that P17 labels the cells in late stage apoptosis by double staining with different stage markers, unveiling that HSP60 may be involved with late stage of apoptosis. Overall, this study has demonstrated that P17 is a novel apoptosis probe targeting HSP60 and promising for the detection of apoptosis *in vitro* and *in vivo*.

## Introduction

Apoptosis is a critical biological process, not only occurring normally during development and aging,^1^ but also having important roles in pathological processes. The maintenance of apoptosis is vital, for example, too much loss of essential cells in the heart may lead to cardiovascular diseases, such as heart failure,^2, 3^ whereas too little apoptosis can give rise to hyperplasia,^4^ cancer^5^ and other morbidities.^6, 7^ Therefore, apoptosis has been developed as one of the important therapeutic strategies.^8, 9^ Thus, identifying cell apoptosis is of great significance to monitoring and assessing the effectiveness of drugs as well as to further understanding the mechanism of apoptosis.

It has been demonstrated that apoptosis can be recognized by several common features, such as phosphatidylserine (PS) exposure on the outer leaflet of cell membrane, caspase activation and the overexpression of heat shock protein (HSP) family members including HSP27, HSP60, HSP70 and HSP90.^10,11,12^ It may be noted that although the function of HSP27, HSP70 and HSP90 have been demonstrated to promote survival of cells upon an apoptotic stimulus, the function of HSP60 is still not fully clarified and both pro-apoptotic or anti-apoptotic has been reported in literature.^11, 13, 14^

To date, prevalent strategies for apoptosis imaging, especially *in vivo*, have been developed on the basis of two apoptotic features, PS exposure and caspase activation, as summarized in detail in the review by Chen *et al.*^15^ In brief, the annexin V is a protein bound to PS and widely used *in vitro* as a marker for apoptosis in all stages. It has been reported that PS may also be exposed on normal cells, such as activated B and T lymphocytes, mast cells and vascular smooth muscle cells.^16, 17^ The 99mTc-annexin-V was the first one to be tested for clinical applications,^16^ whereas as a protein-based probe, annexin V, has slow clearance which reduces the signal-to-noise ratio.^18^ To overcome these limitations, substitutes for annexin V, such as the C2A domain of synaptotagmin I^19, 20^ and PS-targeting peptides,^21, 22^ have been investigated. Another distinctive target for apoptosis probing is caspase. Several substrate-based probes have been developed.^23,24,25^ However, there is a risk that the sequences can also be recognized by other cysteine proteases highly expressed in multiplex organs and tissues.^23^ As for the inhibitor-based caspase probes, most of them are derived from isatin sulfonamide analogs and expected for uses in PET.^26, 27, 28, 29^ Probes of low molecular weight are being investigated intensively owing to their greater feasibilities with radio-labeling procedures^30^ or chemical modification with other imaging agents^15, 31, 32^ than large protein-based probes, so far only ML-10^32, 33^ successfully entered the Phase II clinical trial (NCT00791063) for detection of neurovascular cell apoptosis in brain metastases in response to radiotherapy. Therefore, apoptosis probes to new targets and with low molecular weight are keenly needed both in basic research and in clinical applications.

Herein, we report that a synthetic peptide with a sequence composed of 39 amino residues (named P17) can accumulate dramatically in cells treated with apoptosis inducers. As the result, multiple apoptotic cell lines can be labeled *in vitro* or in the subcutaneous tumor mass of mice. It is shown that P17 can specifically bind to the HSP60 that accumulates in apoptotic cells. We also showed that P17 was likely to label the apoptotic cells in the late stage, unveiling HSP60 was closely involved with late apoptosis.

## Results

### P17 labels TRAIL-induced apoptotic Jurkat cell line

**Figure 1:**
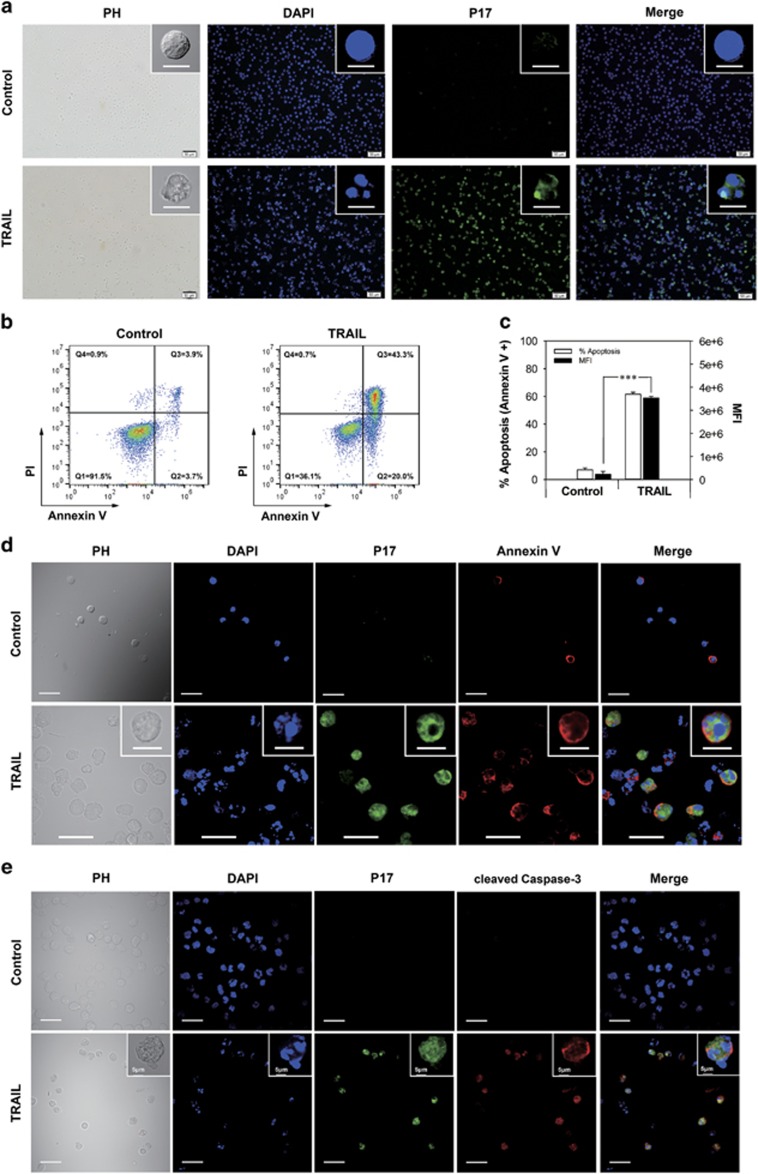
P17 binding and apoptosis analysis in Jurkat cell line after apoptosis induction. (**a**) Fluorescent microscopy observation of extremely large uptake of FITC-P17 (green) by TRAIL-treated Jurkat cell line. Nuclei stained blue with DAPI. Scale bars, 10 μm (top); 50 μm (bottom). (**b**) Apoptosis analysis of the same sample used for P17 binding by double staining with annexin V and PI. The quadrants Q were defined as Q1=live (Annexin V- and PI-negative), Q2=early stage of apoptosis (Annexin V-positive/PI-negative), Q3= late stage of apoptosis (Annexin V- and PI-positive) and Q4=necrosis (Annexin V-negative/PI-positive). (**c**) Flow cytometry quantification of the cellular fluorescence in Jurkat cells treated with TRAIL following incubated with FITC-P17 versus control samples shown via mean fluorescence intensity. Percentage of apoptosis was determined using Annexin V/PI staining and expressed as the proportion of annexin V-positive cell counts in total cell populations. Data are presented as means±s.d. (*n*=3). ***P<0.001. (**d**, **e**) FITC-P17 (green), DAPI (blue) and Annexin V (red; **d**) or cleaved caspase-3 antibody (red; **e**) were colocalized within the same Jurkat cell after treatment with TRAIL to induce apoptosis by confocal microscopy. Scale bars, 10 μm (inserts in **d**); 30 μm (bottom).

In the first section of experiments, we investigated the binding behavior of P17 to Jurkat leukemia T-cell line undergoing drug-induced apoptosis. When TRAIL (TNF-related apoptosis-inducing ligand)-treated Jurkat cells were incubated with fluorescein isothiocyanate (FITC)-P17 at a concentration of 10 μM for 30 min, a palpable fluorescence accumulation was observed in the cells by confocal microscopy while little fluorescence was detected in untreated cells as control (Figure 1a). It was discernible that the TRAIL-treated cells exhibit typical apoptotic morphology, such as plasma membrane retraction and nuclear and cytoplasmic condensation.^34^ To verify the occurrence of apoptosis, Annexin V/Propidium Iodide (PI) staining was applied and the apoptosis proportion of the TRAIL-treated and control cells was 63.3% and 7.6%, respectively (Figure 1b), measured by flow cytometry. Meanwhile, the TRAIL treatment induced an extremely significant increase in the mean fluorescence intensity of the cells compared with the control, indicating a high level of P17 uptake (Figure 1c). To further define that the cells with accumulation of P17 were undergoing apoptosis, annexin V and cleaved caspase-3 antibody (CC3), the two most widely used apoptosis detector *in vitro*, were used for colocalization assay with P17. The confocal observation showed that only annexin V- or CC3-positive cells were labeled with dense P17 (Figures 1d and e).

**Figure 2:**
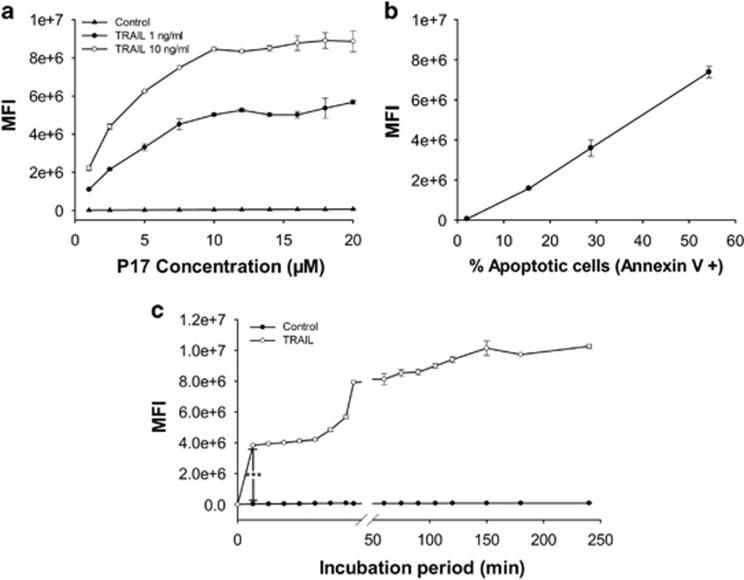
Binding characteristics of P17 to apoptotic cells in concentration or apoptosis degree dependence. All the samples were analyzed by flow cytometry. (**a**) Concentration-dependent uptake of P17 into Jurkat cells with different apoptosis degree. (**b**) A proportional correlation between the apoptosis degree and P17 binding amount in TRAIL-treated cells. The apoptosis degree was determined using Annexin V/PI staining and expressed as the proportion of Annexin V-positive cell counts in total counts. (**c**) Time-course changes of P17 binding amount in TRAIL-treated cells compared to control sample. All results are presented as means±s.d. (*n*=3). ***P<0.001.

We then examined the concentration effect of FITC-P17 on the cellular fluorescent intensity with TRAIL-induced Jurkat cells. As the concentration of FITC-P17 was changed from 1 μM to 20 μM, control cells remained a constant and very low level of fluorescent intensity, while the TRAIL-treated cells showed proportionally elevated fluorescent intensity with the P17 concentration increased and reached the binding platform at 10 μM (Figure 2a). In addition, the fluorescence intensity can be associated to the apoptosis degree within the concentration range of P17: at each tested concentration of P17, the elevated level of apoptosis induced by TRAIL led to increased uptake of P17 in Jurkat cells. Next, Jurkat cells were induced to various degrees of apoptosis through different incubation time with TRAIL and quantified both by FITC-P17 binding as well as by annexin V/PI staining, showing a positive correlation between the binding amount of P17 and the apoptosis degree (Figure 2b). The time course of P17 binding exhibited multiple phases: increased sharply within 2 min of the incubation, remained constant within 2–10 min, and increased fast from 10 min to 15 min followed with the other plateau within 15–60 min of incubation and a slowly elevation within 1–4 h of incubation (Figure 2c). This finding further validated the potential of P17 as an apoptosis probe.

### P17 labels multiple cell lines undergoing apoptosis

**Figure 3:**
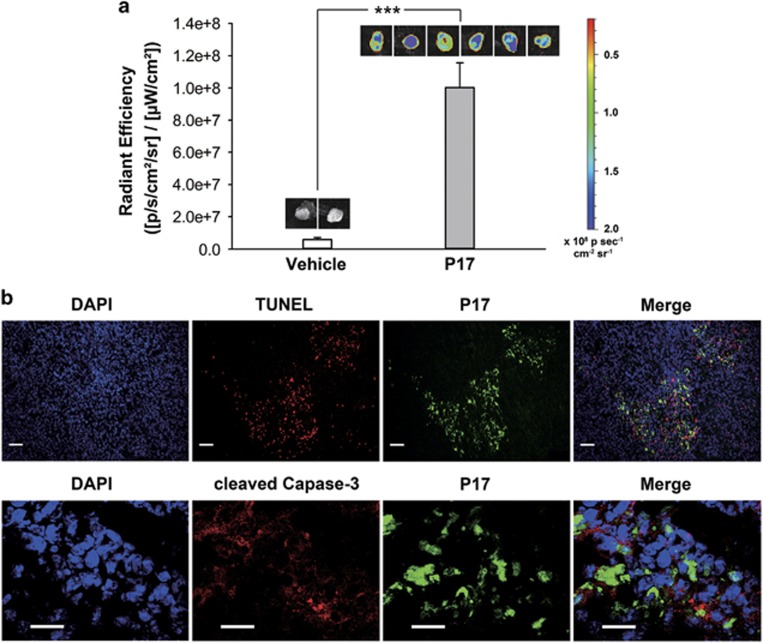
Flow cytometry and confocal microscopy analysis of P17 binding to various cell lines undergoing drug-induced apoptosis. (**a**) Flow cytometry quantification of the cellular fluorescence in cells treated with apoptosis inducers following incubation with FITC-P17 versus control samples shown via mean fluorescence intensity. Percentage of apoptosis was determined using Annexin V/PI staining and expressed as the proportion of Annexin V-positive cell counts in total counts. Data are presented as means±s.d. (*n*=3). ***P<0.001. (**b**) Confocal microscopy observation of FITC-P17 (green) accumulating in cells undergoing apoptosis. Nuclei stained blue with DAPI. Scale bars, 50 μm (THP-1); 100 μm (other cell lines).

Next, additional cell lines undergoing apoptosis were tested with FITC-P17, including K-562 (human chronic myelogenous leukemia), MDA-MB-231 (human breast adenocarcinoma), Hela (human cervical adenocarcinoma) and HUVEC (human umbilical vein endothelium) treated with TRAIL, THP-1 (human acute monocytic leukemia) and DU4475 (human breast carcinoma) treated with camptothecin, and H9c2 (rat myocardium) treated with hydrogen peroxide, in the attempt to demonstrate the potency of P17 as an *in vitro* apoptosis-imaging probe. With a distinct increase of apoptosis degree defined by annexin V/PI staining, the uptake of P17 was elevated by more than 10-fold in mean fluorescence intensity in the cell lines compared with untreated cells as control, except for that by THP-1 which only had a triple increase, partly because the percentage of apoptosis for THP-1 did not change greatly (Figure 3a). Observations with confocal microscopy showed that there was bright fluorescence in the tested cell lines with apoptosis induction, whereas untreated cells as control showed minimum fluorescence (Figure 3b). These findings together demonstrated that P17 can clearly bind to the cells with apoptotic features of annexin V/PI binding and caspase-3 activation.

### Apoptosis imaging in tumors of intravenously injected P17

**Figure 4:**
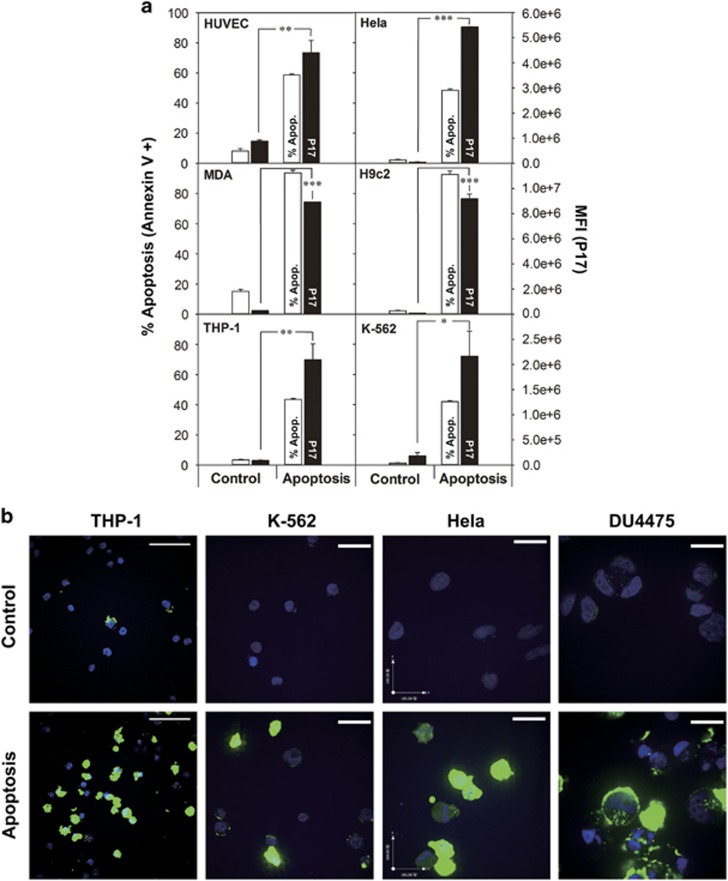
Apoptosis imaging in mouse tumor by P17. (**a**) Imaging and quantification of total FITC fluorescence in excised 4T1 tumors from mice treated with FITC-P17 for 1 h. Data are presented as means±s.d. *P<0.05, **P<0.01, ***P<0.001. (**b**) Histological analysis of apoptosis region in tumor tissues. The excised tumor masses were processed for TUNEL (DNA fragmentation) and cleaved caspase-3 staining after imaging. Green indicated P17; red indicated TUNEL and cleaved caspase-3; blue indicated DAPI for nuclei detection. Scale bars, 50 μm (TUNEL); 20 μm (CC3).

To explore the indicative potential of P17 to the apoptosis *in vivo*, BALB/c mice were subcutaneously injected with murine breast cancer 4T1 cell line. After 8 days, a palpable tumor was developed. Then, the tumor-bearing mice were injected with FITC-P17 by tail vein. After 60 min of injection, the mice were killed and the tumor masses were excised for *ex vivo* fluorescent imaging immediately. Strikingly, P17 showed a significant accumulation in the tumor mass compared with the vehicle-treated group (Figure 4a). Moreover, the excised tumor masses showing bright fluorescence in *ex vivo* imaging were further processed for terminal deoxynucleotidyl transferase dUTP nick end labeling (TUNEL; DNA fragmentation) and cleaved caspase-3 staining commonly used for labeling apoptosis in histology analysis (Figure 4b). It was found that P17 approximately colocalized with TUNEL and cleaved caspase-3 in the same region of tumor mass, confirming the fluorescence signal that was observed by *ex vivo* imaging labeled the apoptotic region. These results strongly suggest that P17 is of great potential for apoptosis detection *in vivo*.

### P17 binds to HSP60 in the cytoplasma

**Figure 5:**
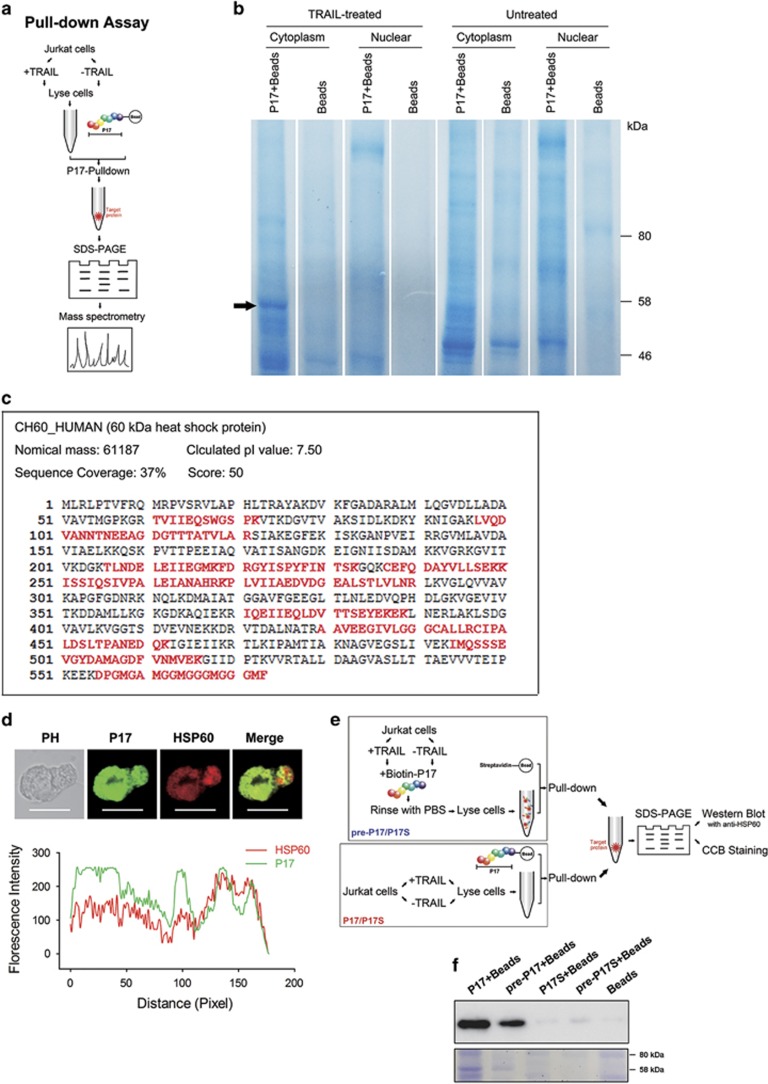
Identification of HSP60 as the target of P17. (**a**) Protocols used for pull-down-assay-based analysis of binder of P17. (**b**) Biotin-P17 labeled streptavidin magnetic beads were used for pull-down assay from TRAIL-treated Jurkat cell extracts. Bound proteins were separated by SDS–PAGE. Absent from TRAIL-untreated and bead-control samples, a major band at 58 kDa was excised for mass spectrometry and identified as HSP60. (**c**) Mass spectrometry analysis identified peptides in HSP60 with 37% sequence coverage. Matched amino acids were shown in red. (**d**) Colocalization of FITC-P17 and anti-HSP60 (Alexa Fluor 594-conjugated) in TRAIL-treated Jurkat cells (Scale bars: 10 μm) observed by confocal microscopy (upper) and analyzed by Image-Pro Plus (bottom). (**e**) The schematic illustration for pull-down and western blot analysis. (**f**) Detection of HSP60 in pull-down samples by western blot and coomassie brilliant blue staining.

On the basis of the confocal microscopy observation (Figures 1d and 3b), we found that P17 localized in the cytoplasm of apoptotic cells. As there were several cytoplasmic proteins with obvious upregulation during apoptosis, including Bax, p53, cleaved caspase-3 and HSPs,^35^ P17 might bind to one of the apoptosis-related proteins mentioned above. To identify the specific binding partners of P17, the pull-down assay was used in TRAIL-treated Jurkat cells (Figure 5a). Absent from TRAIL-untreated or bead-control sample, a specific protein band was observed in the sample of TRAIL-treated cells (Figure 5b). By mass spectrometry analysis, the P17-binding protein was identified to be HSP60 with 37% sequence coverage, a mitochondrial chaperonin accumulating in the cytoplasm during apoptosis (Figure 5c). Some unexpected bands were also observed in TRAIL-treated samples. We considered that in P17 there might be some segments that were possibly bound with other cytoplasmic proteins. It could be noticed that these unexpected bands were also observed in the TRAIL-untreated sample, while the HSP60 band was observed only in the TRAIL-treated sample, which suggested that the unspecified bands are not directly relevant with the cell apoptosis. These bands are worth investigating in our future effort.

To further confirm the interaction between P17 and HSP60, we synthesized a scrambled P17 (named P17S) and used it as a control in two pull-down assays. One protocol is the same as that used for the mass spectrometry analysis; biotinylated P17 was incubated with streptavidin-beads to obtain P17-conjugated beads that was subsequently used to catch the target protein. The other protocol is to pre-incubate biotinylated P17 with TRAIL-treated cells, and then streptavidin-conjugated beads were used to catch the biotin-P17-protein complex. These two protocols have been schematically illustrated in Figure 5e. Results showed that in both protocols, P17S could not bind HSP60 in the apoptotic cells, while P17 bound to HSP60 clearly (Figure 5f).

This result was further confirmed by colocalization with anti-HSP60 antibody. After treatment with TRAIL, Jurkat cells were incubated with FITC-P17 and then stained with anti-HSP60 antibody. It was observed in confocal microscopy that P17 and anti-HSP60 antibody were approximately localized in the same region in the cytoplasm (Figure 5d), indicating the P17 accumulation in apoptotic cells by binding with HSP60.

### P17 is likely to label late stage apoptosis

**Figure 6:**
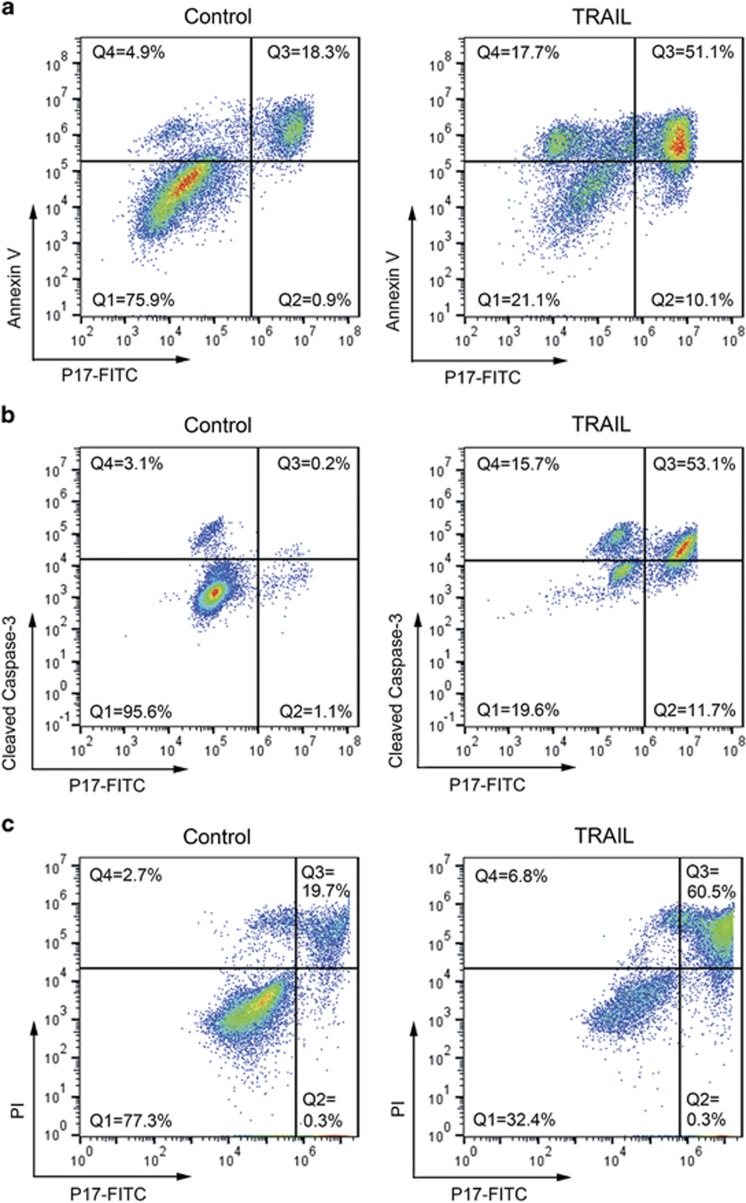
Comparison of P17 with conventional apoptosis detection agents in apoptotic cells. TRAIL-treated and control Jurkat cells were incubated with FITC-P17 for 30 min, and then co-stained with Annexin V (Alexa Fluor 647-conjugated; **a**), cleaved caspase-3 antibody (Alexa Fluor 647-conjugated; **b**) or PI (**c**), before analysis on the flow cytometer. All the samples were plotted as P17 on the *x* axis versus other apoptosis-imaging agents' fluorescence on the *y* axis. The quadrants Q in (**a**) and (**b**) were defined as Q1=live (P17-negative/Annexin V- or CC3-negative), Q2=necrosis (P17-positive/Annexin V- or CC3-negative), Q3=late apoptosis (P17-negative/Annexin V- or CC3-positive) and Q4=early apoptosis (P17-positive/Annexin V- or CC3-negative). The quadrants in (**c**) were defined as Q1=live (P17-negative/PI-negative) and Q3=late apoptosis (P17-positive/PI-positive).

Annexin V is used to detect exposed PS on the outer membrane, and the anti-cleaved caspase-3 (CC3) antibody is marked as the activation of caspase enzyme; both are markers for the whole process of apoptosis, from early to late stages, while PI is a marker to label the late apoptosis. To further characterize the preference of P17 to apoptotic cells in different stages, double staining assays were performed on Jurkat cells with or without TRAIL treatment. As shown in Figure 6a, three distinct cell populations were defined in the flow cytometry chart after TRAIL treatment: P17(−)/Annexin V(−) in Q1, P17(+)/Annexin V(+) in Q3 and P17(−)/Annexin V(+) in Q4. Compared with control, the Q1 cells in the TRAIL-treated group decreased from 75.9% to 21.1%, while the Q3 and Q4 cells increased from 18.3% to 51.1% and from 4.9% to 17.7%, respectively. By P17/CC3 double staining, an increase in Q3 (from 0.2 to 53.1%) and Q4 (from 3.1 to 15.7%), and a decrease in Q1 (from 95.6 to 19.6%) after TRAIL treatment were observed as well (Figure 6b). Taken together, P17 can label a sub-population in Annexin V-positive or CC3-positive cells. To identify the sub-population, a double staining assay of PI/P17 was further conducted. Two major cell populations were observed in the flow cytometry chart (Figure 6c): TRAIL treatment induced an increase of Q3 cells, from 19.7 to 60.5%, and a decrease in Q1 cells, from 77.3 to 32.4%. As most of PI-positive cells were labeled with P17, we therefore inferred that P17 has notable prominence to label cells in the late stage of apoptosis. Moreover, this result unveiled that HSP60 was involved with the apoptosis of late stage.

## Discussion

Probes for apoptosis imaging have been attractive and of significant values for the evaluation of cancer therapy and cytotoxicity monitoring for therapeutics *in vitro* or *in vivo* as well as for drug screening. So far, several noninvasive apoptosis detection strategies have been developed on the basis of targeting specific features during apoptosis as introduced before.^19, 20, 21, 22, 23, 24, 25, 26, 27, 28, 29, 32, 33^ Among these, HSPs have been suggested as the proteins involved in apoptosis process^10,11,12^ and yet to be explored as markers for cell apoptosis. A HSP60-targeting apoptosis detector, P17, is identified in the current study.

Of note, HSP60 has been shown to have apoptosis-promoting function by facilitating the maturation of cleaved caspase-3.^10, 11, 12, 13, 36^ On the other hand, several studies support HSP60's survival-promoting function by finding that HSP60 could be complexed with Bax^11, 12, 37^ or p53^14^ to inhibit their pro-apoptosis functions, or interacted with mitochondria Lon^35^ to sustain its anti-apoptosis function. Despite the complexity of the roles HSP60 has in apoptosis process, data exist supporting an apparent accumulation of HSP60 in the cytoplasm of Jurkat,^10, 36^ Hela,^38^ MDA-MB-231^14^ and cardiac myocytes^37^ during apoptosis. By coincidence, these four cell lines have also been tested in our study and showed an extremely high binding of P17 after apoptosis induction (Figures 1 and 3), further confirming the binding between P17 and HSP60. Interestingly, we find that P17 tends to label cells in the late stage of apoptosis by double staining with two early-to-late apoptosis markers (annexin V and cleaved caspase-3 antibody) and one late stage marker (PI). Hence, it is rational to conclude that HSP60 is involved in the late stage of apoptosis. Our findings may offer a new perspective that would help to understand the mechanism of HSP60 during apoptosis.

Another attractive feature of P17 is that it has high affinity to apoptotic cells. In the cell lines that we have tested, apoptotic Jurkat, Hela, K-562, MDA-MB-231, HUVEC and H9c2 cells showed a strong fluorescent signal 10-fold greater than that of the viable control because of FITC-P17 binding; while with annexin V, apoptosis signal is only two times higher than that of normal cells. This performance makes it much easier to distinguish apoptotic cells from living cells. Importantly, P17 shows great apoptosis-binding ability not only in multiple human tumor cells, but also in human endothelial cell and rat myocardium cells, which suggests that P17 can be used for apoptosis detection not only in cancers but also in cardiovascular diseases.

As a low molecular weight peptide, P17 has some merits over protein-based probes. First, P17 is chemically synthesized, which makes it more suitable to be modified with fluorescent or radioactive groups for clinical applications and more economically to be produced.^2^ In addition, specific buffer and the excess washing processes are required for using annexin V and TUNEL, which may cause unnecessary damages to cells and lead to false-positive results. Differently, P17 can be used directly in conventional culture medium. More importantly, in our *ex vivo* imaging experiment, we found that the fluorescent signal of P17 dropped to control level within 6 h in liver and kidney in mouse model (data not shown), implying that P17 possibly has rapid distribution and clearance *in vivo*. This performance is very necessary when P17 is intended to be used as the imaging marker, because low clearance *in vivo* leads to high background signals and therefore limits success in clinical uses.^39, 40^ Nevertheless, the pharmacokinetics of P17 requires further investigation in further studies.

In summary, P17 has the ability of labeling multiple kinds of apoptotic cells *in vitro* and apoptosis *in vivo* through binding with HSP60 in cytoplasm, exhibiting promising potentials for uses in the visualization of apoptosis in the biomedical researches and applications.

## Materials and methods

### Peptide P17

In the current study, we tested a collection of chemically synthesized peptides based on the fragments of various proteins which were assembled in our previous studies for unraveling various interaction, hydrogen bond, electrostatic and hydrophobic interactions between side chains, in the peptide assemblies using scanning probe microscopy.^41, 42^ Cell-based selection process was performed for screening of the peptide ligands with high binding affinity. A peptide P17 (GDQNLQGPMLQGDPGFQRCIDGNVRLVFLFRGKKKKKKG) was identified by the cell-based selection. For the further verification of P17, a scrambled P17 (P17S) was synthesized (GNLDQMQGLGDPQPGRQCFIVRLFGDNLFRVGKKKKKKG). The molecular weight of P17 or P17S is 4375.14 Da. The N terminal of P17 or P17S was conjugated with FITC or biotin according to the requirement of experiments. The molecular weight of FITC-P17 is 4875.25 Da and the molecular weight of Biotin-P17/P17S is 4601.48 Da. Peptides used in this study were purchased from GL Biochem (Shanghai) Ltd. (Shanghai, China). P17 or P17S is stored at −20 °C. Before use, the peptides were defrosted at room temperature. For cell-based binding assays, P17 or P17S was dissolved in the culture medium at indicated concentrations. For *in vivo* experiments, P17 was dissolved in saline at indicated concentrations.

### Cell culture

All the cell lines mentioned above, except H9c2 and HUVEC, were purchased from the Cell Resource Center of Chinese Academy of Medical Sciences (Beijing, China). H9c2 rat myocardium cell line was kindly provided by Professor Youyi Zhang, Institute of Vascular Medicine, Peking University, Third Hospital (Beijing, China). HUVEC primary human umbilical vein endothelial cell line was purchased from ScienCell Research Laboratories (San Diego, CA, USA). Jurkat T acute lymphoblastic leukemia cells, THP-1 acute monocytic leukemia cells, K-562 chronic myelogenous leukemia cells, DU4475 breast cancer cells, Hela cervical carcinoma cells, H9c2 rat myocardium cells and MDA-MB-231 breast cancer cells were grown in RPMI 1640 (Hyclone Thermo Scientific), DMEM/High glucose (Hyclone Thermo Scientific, Logan, UT, USA) or Leibovitz's L-15 (Gibco by Life Technologies, Carlsbad, CA, USA) containing 10% fetal bovine serum (Gibco by Life Technologies), 100 U/ml penicillin and 100 U/ml streptomycin at 37 °C and 5% CO_2_. 4T1 mice breast cancer cells were cultured in RPMI 1640 containing 10% fetal bovine serum and antibiotics, supplemented with sodium pyruvate, L-glutamine and HEPES at 37 °C and 5% CO_2_. HUVECs were cultured in endothelial cell medium (ScienCell Research Laboratories) in the presence of 5% fetal bovine serum, penicillin/streptomycin and endothelial cell growth supplements in a dilution of 1:5 (ScienCell Research Laboratories) at 37 °C and 5% CO_2_.

### Apoptosis induction and measurement

Jurkat, K-562, Hela and MDA-MB-231 cells were treated with TRAIL (R&D System, Minneapolis, MN, USA) for 24 h at a concentration of 10 ng/ml, 100 ng/ml, 100 ng/ml and 10 ng/ml, respectively. THP-1 and DU4475 cells were incubated with 10-hydroxy camptothecin for 24 h at a concentration of 40 nmol/ml and 80 nmol/ml. Apoptosis of H9c2 cells was induced by hydrogen peroxide at 1.2 mM for 4 h.

To monitor the apoptosis, FITC-Annexin V/PI staining (eBioscience, Vienna, Austria) was used according to the manufacturer's protocol. Briefly, after treatment with apoptosis inducers, cells were harvested and washed with cold phosphate-buffered saline (PBS), and then resuspended in 1 × binding buffer. FITC-Annexin V and PI were added to the cell suspension, followed by incubation at room temperature for 10 min. As soon as possible, stained cells were subjected to flow cytometry, measuring the fluorescence in FL1 channel (FITC) and FL2 channel (PI). Data were analyzed using CFLow Plus (Accuri Cytometers, Ann Arbor, MI, USA) or FlowJo software (Tree Star, San Carlos, CA, USA). All experiments were performed in triplicate.

### Flow cytometry and confocal microscopy analysis of P17 binding to apoptotic cells

In flow cytometry (FCM) analysis, 5 × 10^5^ (for Jurkat and THP-1), 2 × 10^5^ (for K-562), 1 × 10^5^ (for Hela), 3 × 10^4^ (for MDA-MB-231), 4 × 10^4^ (for H9c2) and 1.5 × 10^5^ (for HUVEC) cells were seeded in 24-well plates in complete growth medium. After 4-h (for suspension cells) or 24- h (for adherent cells) pre-culture, apoptosis inducers were added and incubation continued for specified periods as described above. Then, FITC-P17 at a concentration of 10 μM was directly added into the growth medium and cells were incubated for a further 30 min at 37 °C. After washing with cold 1 × PBS two times, cells were subjected to flow cytometry analysis by C6 Accuri (Accuri Cytometers). Data were analyzed using CFLow Plus (Accuri Cytometers) or FlowJo software (Tree Star). All experiments were performed in triplicate.

For fluorescence imaging, 1 × 10^5^ Hela cells were plated on glass coverslips, while suspension cells (Jurkat, THP-1, K-562 and DU4475) were cultured as described above. After treatment with apoptosis inducers, cells were incubated with FITC-P17 for a further 30 min at 37 °C. Then, the cells were fixed in 4% paraformaldehyde for 15 min at 37 °C, followed by rinsing with cold PBS two times. The resulting cells were mounted on a glass slide using an aqueous mounting medium containing DAPI as nuclear stain (Zhongshan Goldenbridge Biotechnology Co., Beijing, China). Images were taken using the Olympus FV1000 Inverted Confocal IX81 Microscope (FV1000+IX81; Olympus America Inc., Center Valley, PA, USA) and analyzed by FV10-ASW software (Olympus) or the UltraVIEWVoX spinning disk confocal microscope (Perkin Elmer, Waltham, MA, USA) and analyzed by Volocity-5 software (Perkin Elmer), at an excitation wavelength of 488 nm for FITC and of 405 nm for DAPI.

### Characterization for P17-binding versus incubation time and concentration

Jurkat cells were seeded in 24-well plates at 5 × 10^5^ cells per well. In the dose- and time-effect assay, after treatment with TRAIL (10 ng/ml) for 24 h, the cells were incubated with FITC-P17 at different concentrations (0–20 μM) for 30 min, or at a concentration of 10 μM for indicated time (0–4 h), respectively. In the apoptosis degree-dependent binding assay, Jurkat cells were treated with TRAIL (10 ng/ml) for different time (0 h, 6 h, 12 h and 24 h), followed by incubation with FITC-P17 (10 μM) for 30 min. Cells were then washed with PBS and subjected to flow cytometry analysis by C6 Accuri. Data were analyzed using CFLow Plus.

### Colocalization of P17 and other apoptosis detection agents

Jurkat cells were seeded in 24-well plates at 5 × 10^5^ cells per well. After treatment with TRAIL (10 ng/ml) for 24 h, the cells were incubated with FITC-P17 (10 μM) for a further 30 min at 37 °C. Next, the cells were collected and washed with cold PBS two times. Colocalizaiton was performed by using Alexa Fluor 647-Annexin V (Invitrogen Life Technologies, Gaithersburg, MD, USA) or Alexa Fluor 647-Cleaved Caspase-3 mAb (#9602; Cell Signaling Technology, Beverly, MA, USA), according to the manufacturers' instructions. Briefly, for annexin V staining, the cells were resuspended in binding buffer (10 mM HEPES, 140 mMNaCl and 2.5 mM CaCl2, pH 7.4), followed by addiction of Alexa Fluor 647-Annexin V and incubation at room temperature for 15 min. For cleaved caspase-3 labeling, the cells were first fixed in 4% paraformaldehyde for 10 min at 37 °C, and then permeabilized in ice-cold 90% methanol for 30 min on ice, followed by rinsing with 0.5% bovine serum albumin/PBS two times. Next, the cells were incubated with rabbit monoclonal anti-human cleaved caspase-3 antibody in bovine serum albumin/PBS for 1 h at room temperature. All the slides were mounted using an aqueous mounting medium containing DAPI as nuclear stain. Images were taken using the Olympus FV1000 Inverted Confocal IX81 Microscope (FV1000+IX81; Olympus America Inc.,) and analyzed by FV10-ASW software (Olympus) at an excitation wavelength of 635 nm for Alexa Fluor 647, 488 nm for FITC and 405 nm for DAPI.

### Cytoplasmic and nuclear protein extraction, P17 pull-down assay and protein identification

Jurkat cells (1 × 10^7^) were induced with TRAIL at 10 ng/ml for 24 h to undergo apoptosis. After washing with cold PBS two times, cytoplasmic and nuclear protein extracts were prepared with NE-PER Nuclear and Cytoplasmic Extraction Reagents kit (Thermo Fisher Scientific, Waltham, MA, USA), according to the manufacturer's protocol. The protein extracts were maintained on ice before downstream processing. To identify the binding partners of P17, pull-down assay was used as described previously.^43, 44^ Briefly, streptavidin magnetic beads (New England Biolabs, Beverly, MA, USA) were incubated with biotinylated P17 in binding buffer (0.5 M NaCl, 20 mM Tris-HCl, 1 mM EDTA, pH 7.5) at 4 °C for 2 h and vortexed several times. Unbound P17 were removed by washing two times in binding buffer. Approximately 75 μg of P17 was used per pull-down. Protein samples that had been precleared with the magnetic beads were mixed with P17-beads in binding buffer on ice for 4 h and the mixture was vortexed every half hour. Then, the supernatant was removed by using a magnet and the beads were washed three times with binding buffer. Boiled in 1 × protein-loading buffer (TransGen Biotech, Beijing, China), the bound proteins were eluted from the beads. Eluates were subjected to SDS–PAGE and stained with coomassie brilliant blue R250.

The specific protein band was excised from the stained SDS–PAGE gel and analyzed by mass spectrometry performed by Professor Zhili Li's Laboratory in the Department of Biophysics and Structural Biology at Institute of Basic Medical Sciences, Chinese Academy of Medical Sciences. Briefly, after the excision, the stripe of SDS–PAGE gel was digested by trypsin and identified by MS using MALDI-FTICR MS (Bruker Daltonics, Billerica, MA, USA). Precisely, 0.5 μl of the enzymatic hydrolysate was spotted onto the MALDI target plate, mixed with 0.5 μl of saturated solution of α-cyano-4-hydroxycinnamic acid in 38% acetonitrile/0.1% trifluoroacetic acid served as matrix. The peptide calibration standard II mixture (Bruker Daltonics) was used to calibrate the instrument with a resolution of 530 000 at m/z 400 over the m/z range of 800–4000 in positive ion mode. The peptide list files were generated using Data Analysis 4.0 software (Bruker Daltonics) and searched against a concatenated Swiss-Prot database and NCBInr human database.

### Pull-down assay and western blot analysis

Jurkat cells (1 × 10^7^) were treated with TRAIL at 10 ng/ml for 24 h to undergo apoptosis. For the group of pre-P17/P17S+beads, the cells were pre-incubated with biotin-P17 or P17S (10 μM) for 30 min at 37 °C, followed by washing with cold PBS two times. Then, the cells were lysed in RIFA lysis buffer (Beyotime Biotechnology, Haimen, China) containing protease inhibitors (Sigma-Aldrich, St Louis, MO, USA) on ice for 30 min. For the group of P17/P17S+Beads, the TRAIL-treated cells were directly suspended in RIFA to extract the proteins. All the protein extracts were maintained on ice before downstream processing. Streptavidin magnetic beads (New England Biolabs, Beverly, MA, USA) for the 'pre-P17/P17S+beads' group, and P17 or P17S-conjugated beads for 'P17/P17S+beads' group were incubated with the precleared protein samples in binding buffer (0.5 M NaCl, 20 mM Tris-HCl, 1 mM EDTA, pH 7.5) on ice for 4 h. The bound proteins were eluted from the beads by boiling in 1 × protein-loading buffer (TransGen Biotech, Beijing, China). Eluates were subjected to SDS–PAGE.

For western blotting, after electrophoresis and transfer to PVDF membranes (0.2 mm; Millipore, Bedford, MA, USA), HSP60 was detected with monoclonal Ab against HSP60 (ab13532; Abcam, Cambridge, UK) at a dilution of 1:10 000 and corresponding secondary antibody conjugated with HRP (A9917; Sigma-Aldrich) at a dilution of 1:40 000. Antibodies were detected by Immobilon Western Chemiluminescent HRP Substrate (Millipore) and imaged by Image Quant LAS 4000 (GE Healthcare, Waukesha, WI, USA). For coomassie brilliant blue staining, Coomassie Blue Staining R250 Kit (Beyotime Biotechnology) was used, according to the manufacturer's protocol.

### Colocalization of P17 and HSP60 antibody

Jurkat cells were treated with 10 ng/ml TRAIL for apoptosis to occur, following incubation with 10 μM FITC-p17 at 37 °C for 30 min, and then washing with cold PBS two times. After fixation with 100% methanol at −20 °C for 10 min, the cells were permeabilized and blocked in 1% bovine serum albumin in 0.1% PBS-Tween for 1 h at room temperature. Approximately 1 × 10^6^ cells were stained with 5 μl anti-hsp60 antibody (ab13532; Abcam) in 100 μl 1% bovine serum albumin in 0.1% PBS-Tween for 1 h at room temperature. The secondary antibody, Alexa Fluor 594 goat anti-mouse IgG (405326; BioLegend, San Diego, CA, USA), was used at 2.5 μg/ml for 1 h. Resulting cells were mounted using an aqueous mounting medium. Images were taken using the Olympus FV1000 Inverted Confocal IX81 Microscope (FV1000+IX81; Olympus America Inc.) and analyzed by FV10-ASW software (Olympus) at an excitation wavelength of 559 nm for Alexa Fluor 549 and 488 nm for FITC. The colocalization of merged images was analyzed by Image-Pro Plus (Media Cybernetics, Rockville, MD, USA).

### *Ex vivo* and histologic analysis of apoptosis in mice tumors

All the animal experiments reported below were approved by the committee on the Animal Care and Use of Institute of Basic Medical Sciences, Chinese Academy of Medical Sciences & Peking Union Medical College (Beijing, China), and followed their approved guidelines. Six-week-old female BALB/c mice were obtained and housed in the Experimental Animal Center at the Institute of Basic Medical Sciences, Chinese Academy of Medical Sciences. All the mice were injected with 4T1 mouse breast cancer cells subcutaneously. After 8 days, the implanted cells had developed into a palpable tumor. Then, the mice were randomly divided into vehicle group (*n*=2) and P17 group (*n*=6), followed by intravenous injection with 100 μl 0.9% (wt/vol) sodium chloride (Shandong Hualu Pharmaceutical Co. Ltd., Beijing, China) and fluorescent P17 (50 nmol) in 100 μl 0.9% (wt/vol) sodium chloride, respectively. After 1 h, the tumor mass was removed and immediately imaged by Xenogen IVIS Spectrum system (Caliper life science, Hopkinton, MA, USA) and analyzed by Living Image software (Xenogen).

For histology, the tumors were directly embedded into OCT compound (Sakura Finetek, Torrance, CA, USA), quickly frozen in liquid nitrogen, and then sectioned at a thickness of 10 μm. Frozen sections were stored at −80 °C until use. Detection of apoptosis in tumor sections was performed by TUNEL (In Situ Cell Death Detection Kit, TMR red; Roche Diagnostics GmbH, Mannheim, Germany) and cleaved-Caspase-3 apoptosis assay, according to the manufacturers' instructions. Briefly, for TUNEL assay, frozen sections were fixed in 4% paraformaldehyde and permeabilized in 0.1% Triton X-100, 0.1% sodium citrate, followed by labeling with enzyme solution (terminal deoxynucleotidyl transferase) and label solution (nucleotide) subsequently. The resulting sections were mounted with an aqueous mounting medium containing DAPI as nuclear stain. Images were taken by a fluorescence microscope (Olympus Optical Co. Ltd, Tokyo, Japan). For cleaved-Caspase-3 assay, after fixation in 3% paraformaldehyde and permeabilization in 10% H_2_O_2_ (30%) in methanol, cryostat sections were stained with cleaved-caspase-3 rabbit mAb (#9664; Cell Signaling Technology) followed by Alexa Fluor 594-secondary antibody (R37119; Invitrogen Life Technologies). Mounted with an aqueous mounting medium containing DAPI, the sections were imaged by the Olympus FV1000 Inverted Confocal IX81 Microscope. All the processes mentioned above should be protected from light.

### Statistical analyses

Data were presented as means±s.d. Statistical significance of the results were analyzed by Student's *t*-test (two-sided), and defined as significant (*, 0.01<*P*<0.05), very significant (**, 0.001<*P*<0.01) and extremely significant (***, *P*<0.001).
